# Supplementary material for: “It doesn’t require blood!”: Perceptions around non-invasive malaria testing tools in Indonesia, Peru, and Rwanda
Source: PLoS One. 2026 Jan 9;21(1):e0318393. doi: 10.1371/journal.pone.0318393 (PMC12788683; doi:10.1371/journal.pone.0318393)
Supplement: S1 File — (DOCX) [file pone.0318393.s002.docx]

Appendix: Semi-Structured Interview Guide

**Opening statement**

Thank you for taking the time to talk to me today. My name is __________________, from _________________. We have been mandated to conduct this study by FIND Geneva which is the sponsor of the study.

We asked you to participate in this interview to talk about three new malaria testing tools. We would like to know what you think about them and if in the future, you would agree to use them or not. Your opinion on this issue is very valuable for us. This is the first study asking opinions of people on these tools for malaria diagnosis, so feel free to express all your perceptions and concerns.

Our interview should last approximately 40 minutes to 1 hour. I will start to introduce the new tools and then ask you some specific questions.

Do you have any question?

Before we start, I will ask you to read and sign the consent form in front of you or collect the consent form that has been sent to you.

For the research team:

- Check that the consent forms are signed and a copy has been given to the participant.
- Switch on the recorder

**ICE-BREAKING QUESTION**

Before starting this interview, could you please tell me:

- ***What is / means malaria for you? Could you please tell us, spontaneously, what comes into your mind ?*** Roundtable

**PRESENTATION OF THE DEVICES**

To present the devices and ask the following questions:

- ***What do you think about this tool / these tools?* *Spontaneously tell us what comes into your mind ?*** (List of advantages and disadvantages, first impressions, concerns, emotions, etc.)

**ACCEPTABILITY**

- ***Is this/these new tool(s) (not) acceptable for you?*** *Explain why*
- ***Is this/these new tool(s) (not) appropriate to your country (cultural sensitivity) ?*** *Explain why*

Let’s talk about the performance of the test (s) :

- ***Are you familiar with the concept of “sensitivity and specificity”?*** *If yes ask next question, if not, give the information : Sensitivity tells us how good a test is at correctly identifying people or cases who have a certain condition or disease. In this case if a test has a sensitivity of 90%, it means that the test can correct identify 90 out of 100 people who actually have malaria. So, it has a high chance of identifying the disease if someone has it. Specificity tells us how good a test is at correctly ruling out people who do not have the condition or the disease. So, in this case a specificity of 90%, means that the test can correctly identify that there are 90 people out of a 100 who do not have malaria.*
- ***Which would be the minimum level of sensitivity that you could accept to trust this test?***
- ***Would you accept the same sensitivity in a healthcare center, compared to a school, or at a border? Could you accept a higher or lower percentage in these different settings? And why?***
- ***Which would be the minimum specificity (negative cases confirmed that they are negative) that you could accept?***
- ***Would you accept the same percentage in a healthcare center compared to a school, or at a border? Could you accept a higher or lower percentage in these different settings ? And Why?***
- ***If you were to be tested now with this/these new tool(s), how would you feel?*** *Check fears, resistance, feelings, emotional barriers etc -> feelings Indonesia, this question might not work*
- ***In which circumstances, this tool/these tools should be used?*** *Raining season, …*
- ***Do you think everyone should use this tool/these tools or only specific groups?*** *If spec, who? If everyone why?*
- ***What do you think are the benefits of this tool/these tools?*** *Collective good (protect all, early diagnosis), individual (prevention, quick treatment, not being sick etc)*
- ***What are the disadvantages of this tool/these tools?*** *Stigma, discrimination, pain….*
- ***Would you recommend it/them to your family or friends?***

**IMPLEMENTATION**

- ***Where would you prefer to see this tool/these tools being implemented*** *(where people shall be tested)* ***?***
- ***If this tool/these tools is/are implemented outside a health care setting how shall it/they be handled?***
- ***Who should be responsible to perform the test(s)? Give the results? Do the counselling?***
- ***Do you think that to be able to use this tool/these tools, a lot of training is needed or everyone could do it?***
- ***If tomorrow this tool / these tools is/are accepted by your government, are you willing to see it/them integrated in the malaria system and used?***
- ***Shall it be mandatory or optional?***
- ***How this new tool could fit in the actual Malaria system?*** *Replace a test, additional test etc.*
- ***Who should be in charge of implementing this new tool ?*** *MOH, schools directors, …*
- ***From your perspective, could it be used for other diseases/conditions?*** *To dig more this answer*
- ***Would any new types of policies need to be enacted to allow for these tools to be used?*** *If so, please give specifics as to the types of policies that would be needed and who would be responsible for introducing these new policies.*

**INFORMATION, STATUS**

- ***From your point of view, who should be in charge of disclosing your malaria status?*** *Only health professionals or it is ok to have a non-health professional disclosing the result such as school teacher, @boarders (who? Guards?)*
- ***Who could know your status? And who couldn’t?*** *Ok to disclose, not ok*
- ***Would you like to keep this information confidential or from your point of view, this information is not sensitive?***

**ENDING QUESTIONS**

We reach the end of our discussion, so before you leave:

- ***Is there anything else you would like to add that is important to consider before integrating and implementing these new samples that we did not cover in this discussion?***
- ***Could you please tell me, why you agree to participate in this interview and study?***
- ***Finally, would you like to be informed about the results of the study?***

**We thank you again for your participation! Ask participant to fill out the socio-demographic questionnaire.**
