# Supplementary material for: “It doesn’t require blood!”: Perceptions around non-invasive malaria testing tools in Indonesia, Peru, and Rwanda
Source: PLoS One. 2026 Jan 9;21(1):e0318393. doi: 10.1371/journal.pone.0318393 (PMC12788683; doi:10.1371/journal.pone.0318393)
Supplement: S2 File — (DOCX) [file pone.0318393.s003.docx]

Appendix : Focus Group Guide

**Opening statement**

Welcome to our focus group discussion and thank you for taking the time to talk to us today.

My name is __________________, from _________________. We have been mandated to conduct this study by FIND Geneva which is the sponsor of the study.

We asked you to participate in this focus group discussion to talk about three new malaria testing tools. We would like to know what you think about them and if in the future, you would agree to use them or not, etc. Your opinion on this issue is important! This is the first study asking point of views of people on these tools, so feel free to express all your concerns. There are some basic things we would like to remind you:

1. We would be asking questions to the group, and anyone could answer. You do not need to follow any specific order, feel free to raise your hand and talk;
2. You can interact with others. Respect others' time while speaking, do not interrupt them, but feel free to talk after another person has finished;
3. There are no good or bad answers, right, or wrong answers. This is not a test, and all responses are valid. Even if you disagree with somebody, please respect their opinion. It is unbelievably valuable for us to know different perceptions and opinions. Do not be afraid to talk and express your perceptions;
4. We also kindly remind you that this discussion is confidential therefore everything that is discussed here, stays here;
5. We might use some of your sentences to illustrate the results of our research. But all quotes will be anonymous. Please, let us know now if anyone has problems with us publishing your quotes.

Our discussion should last approximately one hour and half, without interruptions. We will start to introduce the new tools and then start the discussion. At the end of the discussion we will ask you to fill out some sociodemographic information *[the sociodemographic form can also be completed before the start of the FG, depending on country preferences]* . We can help you to complete this form.

Do you have any question?

Before we start, I will ask you to read and sign the consent form in front of you or collect the consent form that has been sent to you.

For the research team:

- Check that all consent forms are signed and a copy has been given to the participant.
- Switch on the recorder

**ICE-BREAKING QUESTION**

Before starting this conversation, could you please tell us:

- ***What is / means malaria for you? Could you please tell us, spontaneously, what comes into your mind ?*** Roundtable

**PRESENTATION OF THE DEVICES**

To present the three devices together and then ask the following questions:

***What do you think about this tool/these tools?* *Spontaneously tell us what comes into your mind ?*** (List of advantages and disadvantages, first impressions, concerns, emotions, etc.)

**ACCEPTABILITY**

I'll now present one hypothetical scenario/situation in which this tool/these tools could be used, and then we'll discuss it together.

**General scenario:**

Lea and John, aged 5, attend the same school. Their parents have been informed that the school has introduced new prevention rules to fight against malaria. Starting next month, malaria will be tested weekly at the school entrance using this new tool [Name of the tool]. All those wishing to enter the school will be tested, therefore it will be compulsory for children, teachers, school staff, parents, etc. All those tested will know their results on-site, i.e. their malaria status. The parents of John and Lea think it’s a good idea to screen their children, but they are also concerned about this new rule and setting.

- ***What do you think about this situation?***
- ***What are your advises to Lea and John’s parents?***
- ***Is this/these new tool(s) (not) acceptable for you?*** *Explain why*
- ***Is this/these new tool(s) (not) appropriate to your country (cultural sensitivity) ?*** *Explain why*
- ***Is this new rule (not) acceptable for you?*** *Explain why (mandatory or optional)*
- ***If you were to be tested now with this tool /these tools, how would you feel?*** *Check fears, resistance, feelings, emotional barriers etc -> feelings Indonesia, this question might not work*

**This scenario should introduce/cover some questions below -> to use answers to probe the questions below. Scenario to be adapted to country specificities.**

- ***If available, would you use this tool/these tools?*** *Explain why yes or why no*
- ***In which circumstances would you use this tool/these tools?*** *“Only if I’m sick”, “fever only” or as a prevention tool or only during raining season or “only if I’m pregnant”, etc?*
- ***What do you think are the benefits of this tool/these tools?*** *Collective good (protect all, early diagnosis), individual (prevention, quick treatment, not being sick etc)*
- ***What are the disadvantages of this tool/these tools?*** *Stigma, discrimination, pain….*
- ***Would you recommend it/them to your family or friends?*** *Explain why yes, why no. Children ok, or not ok, too young, risk etc ?*

**ADDITIONAL QUESTIONS ON PERFORMANCE ONLY FOR THE FG WITH HEALTHCARE WORKERS:**

- ***Are you familiar with the concept of “sensitivity and specificity”?*** *If yes ask next question, if not, give the information : Sensitivity tells us how good a test is at correctly identifying people or cases who have a certain condition or disease. In this case if a test has a sensitivity of 90%, it means that the test can correct identify 90 out of 100 people who actually have malaria. So, it has a high chance of identifying the disease if someone has it. Specificity tells us how good a test is at correctly ruling out people who do not have the condition or the disease. So, in this case a specificity of 90%, means that the test can correctly identify that there are 90 people out of a 100 who do not have malaria.*
- ***Which would be the minimum level of sensitivity that you could accept to trust this test?***
- ***Would you accept the same sensitivity in a healthcare center, compared to a school, or at a border? Could you accept a higher or lower percentage in these different settings? And why?***
- ***Which would be the minimum specificity (negative cases confirmed that they are negative) that you could accept?***
- ***Would you accept the same percentage in a healthcare center compared to a school, or at a border? Could you accept a higher or lower percentage in these different settings? And Why?***
- ***What are the key characteristics that each instrument shall have?*** *(list key characteristics quoted by each participant and check if the ability to differentiate parasite species emerged. If not, ask the question: Do you think it would be relevant if the instrument can differentiate between parasite species (Vivax, Falciparum, Ovale, Malaria, knowlesi)?*

**IMPLEMENTATION**

- ***Where preferably would you like to use this tool/these tools*** *(where would you like to be tested)****?***
- ***Do you think it/they should be everywhere or only available in specific places?*** *Schools, airports, ….*
- ***Who would benefit the most of using this tool/these tools?*** *Only pregnant women, …*
- ***Do you think everyone should use this tool/these tools or only specific groups?*** *If spec., who? If everyone why?*
- ***Do you think there is a specific time to use this tool/these tools or it/they can be used anytime?*** *Raining seasons, holidays, natal care visits, after another diagnosis?*
- ***Who should be in charge of implementing this new tool/these new tools ?*** *MOH, schools dir.,…*

**INFORMATION, STATUS**

- ***From your point of view, who should be in charge of disclosing your malaria status?*** *Only health professionals or it is ok to have a non-health professional disclosing the result such as school teacher, @boarders (who? Guards?)*
- ***Who could know your status? And who couldn’t?*** *Ok to disclose, not ok*
- ***Would you like to keep this information confidential or from your point of view, this information is not sensitive?***

**ENDING QUESTIONS**

We reach the end of our discussion, so before you leave:

- ***Is there anything else you would like to add that is important to consider before integrating and implementing these new testing tools at we did not cover in this discussion?***
- ***Could you please tell us, why you agree to participate in this focus group and study?***
- ***Finally, would you like to be informed about the results of the study?***

**We thank you again for your participation! Ask participants to fill out the socio-demographic form.**
