# Supplementary material for: “It doesn’t require blood!”: Perceptions around non-invasive malaria testing tools in Indonesia, Peru, and Rwanda
Source: PLoS One. 2026 Jan 9;21(1):e0318393. doi: 10.1371/journal.pone.0318393 (PMC12788683; doi:10.1371/journal.pone.0318393)
Supplement: Attachment — Submitted filename: Response to reviewers.docx [file pone.0318393.s006.docx]

Journal Requirements:

When submitting your revision, we need you to address these additional requirements.

1. Please ensure that your manuscript meets PLOS ONE's style requirements, including those for file naming. The PLOS ONE style templates can be found at

<https://journals.plos.org/plosone/s/file?id=wjVg/PLOSOne_formatting_sample_main_body.pdf> and

[https://journals.plos.org/plosone/s/file?id=ba62/PLOSOne_formatting_sample_title_authors_affiliations.pdf](https://track.editorialmanager.com/CL0/https:%2F%2Fjournals.plos.org%2Fplosone%2Fs%2Ffile%3Fid=ba62%2FPLOSOne_formatting_sample_title_authors_affiliations.pdf/1/010f0197a1ee0a23-7c5f961b-1d73-43b3-bcd4-b30bc4ac3907-000000/snAs-pv9qtdY2YPjIanzeFRGRxp8feVWuZU7ie0bQHQ=216)

 Thank you for the reminder. We reviewed the guidelines and revised the manuscript to ensure it meets the formatting guidelines. Please advise if we missed anything.

2. Please include a complete copy of PLOS’ questionnaire on inclusivity in global research in your revised manuscript. Our policy for research in this area aims to improve transparency in the reporting of research performed outside of researchers’ own country or community. The policy applies to researchers who have travelled to a different country to conduct research, research with Indigenous populations or their lands, and research on cultural artefacts. The questionnaire can also be requested at the journal’s discretion for any other submissions, even if these conditions are not met.  Please find more information on the policy and a link to download a blank copy of the questionnaire here: <https://journals.plos.org/plosone/s/best-practices-in-research-reporting>. Please upload a completed version of your questionnaire as Supporting Information when you resubmit your manuscript.

Thanks, we uploaded the questionnaire as required

3. Thank you for stating the following financial disclosure:
This study has been funded by UK aid from the UK government (FCDO project number 300341-102), as well as the Swiss Development Cooperation (contract number 81071426).

Please state what role the funders took in the study. If the funders had no role, please state: "The funders had no role in study design, data collection and analysis, decision to publish, or preparation of the manuscript."
If this statement is not correct you must amend it as needed.
Please include this amended Role of Funder statement in your cover letter; we will change the online submission form on your behalf.

Thanks for your comment, we added the sentence in the cover letter as required.

4. We noted in your submission details that a portion of your manuscript may have been presented or published elsewhere. Please clarify whether this publication was peer-reviewed and formally published. If this work was previously peer-reviewed and published, in the cover letter please provide the reason that this work does not constitute dual publication and should be included in the current manuscript.

Dear Editor, the publication is now formally published (Nussbaum, L., Ortega, E., Ríos López, E.J. *et al.* Voices from the Amazon: exploring implementor and user perceptions of non-invasive malaria diagnostics in Peru. *Malar J* **24**, 32 (2025). https://doi.org/10.1186/s12936-025-05273-1). As requested, we added a section in the cover letter explaining why this doesn’t constitute dual publication.

5. Please amend the manuscript submission data (via Edit Submission) to include author Claudius Mone, Amy C. Morrison

We have checked the manuscript submission data and both authors Claudius Mone and Amy C. Morrison are listed.

6. Please amend your authorship list in your manuscript file to include author Claudius Novchovick Mone Iye, Amy Celeste Morrison

Thanks, we addressed this change in the manuscript file

7. We notice that your supplementary figures are uploaded with the file type 'Figure'. Please amend the file type to 'Supporting Information'. Please ensure that each Supporting Information file has a legend listed in the manuscript after the references list.

Thank you for bringing this to our attention. The figure has now been correctly uploaded as a Supporting Information file.

8. Please include captions for your Supporting Information files at the end of your manuscript, and update any in-text citations to match accordingly. Please see our Supporting Information guidelines for more information: <http://journals.plos.org/plosone/s/supporting-information>.

Thank you for pointing this out. The Supporting Information captions have been added at the end of the manuscript. We will remove the separate caption file from the submission files, in accordance with the guidelines.

9. Please review your reference list to ensure that it is complete and correct. If you have cited papers that have been retracted, please include the rationale for doing so in the manuscript text, or remove these references and replace them with relevant current references. Any changes to the reference list should be mentioned in the rebuttal letter that accompanies your revised manuscript. If you need to cite a retracted article, indicate the article’s retracted status in the References list and also include a citation and full reference for the retraction notice.

Thank you very much for your comment. We have reviewed the references and none of them seem to have been retracted, please let us know if we missed anything.

Additional Editor Comments:

The reviewers thought that this paper provides valuable information. Please respond carefully to their comments; reviewer 2 has provided detailed constructive comments.


[Note: HTML markup is below. Please do not edit.]

Reviewers' comments:

Reviewer's Responses to Questions

**Comments to the Author**

1. Is the manuscript technically sound, and do the data support the conclusions?

The manuscript must describe a technically sound piece of scientific research with data that supports the conclusions. Experiments must have been conducted rigorously, with appropriate controls, replication, and sample sizes. The conclusions must be drawn appropriately based on the data presented.

Reviewer #1: Yes

Reviewer #2: Yes

2. Has the statistical analysis been performed appropriately and rigorously?

Reviewer #1: N/A

Reviewer #2: N/A

3. Have the authors made all data underlying the findings in their manuscript fully available?

The [PLOS Data policy](http://track.editorialmanager.com/CL0/http:%2F%2Fwww.plosone.org%2Fstatic%2Fpolicies.action%23sharing/1/010f0197a1ee0a23-7c5f961b-1d73-43b3-bcd4-b30bc4ac3907-000000/ldUX-0qdJQ2Ffx2j5vp32OwQJWLZwyAqxL7ODWl1vC8=216) requires authors to make all data underlying the findings described in their manuscript fully available without restriction, with rare exception (please refer to the Data Availability Statement in the manuscript PDF file). The data should be provided as part of the manuscript or its supporting information, or deposited to a public repository. For example, in addition to summary statistics, the data points behind means, medians and variance measures should be available. If there are restrictions on publicly sharing data—e.g. participant privacy or use of data from a third party—those must be specified.

Reviewer #1: Yes

Reviewer #2: No

4. Is the manuscript presented in an intelligible fashion and written in standard English?

PLOS ONE does not copyedit accepted manuscripts, so the language in submitted articles must be clear, correct, and unambiguous. Any typographical or grammatical errors should be corrected at revision, so please note any specific errors here.

Reviewer #1: Yes

Reviewer #2: Yes

5. Review Comments to the Author

Please use the space provided to explain your answers to the questions above. You may also include additional comments for the author, including concerns about dual publication, research ethics, or publication ethics. (Please upload your review as an attachment if it exceeds 20,000 characters)

Reviewer #1: The manuscript is well-written, with correct grammar and proper sentence structure.
The article provides a comprehensive and accurate overview of the current state of malaria diagnostics and the challenges faced. It effectively discusses the limitations of traditional methods and the potential of new technologies that can be used instead. The references are well structured.
The researchers did a good job of explaining complex concepts and providing detailed information about various diagnostic methods. The section on malaria testing tools is informative. It explains the three types of devices, including their current market status and potential applications, which sets up the context for evaluating their acceptability and effectiveness.
Method and material section is well-organized into sections (Study design and sites, Study populations, Sampling procedures, and Malaria testing tools), which help guide the reader through the methodology. Various groups were included (community members, pregnant women, teachers, caregivers, HCWs, border professionals, and stakeholders). This variety supports a rich understanding of different perspectives regarding malaria diagnostics, especially in endemic areas.
Discussion section does an excellent job of capturing a wide range of perspectives on non-invasive malaria diagnostics and demonstrates the potential value of these tools in improving malaria screening and treatment in diverse settings. The text provides a thorough exploration of both the advantages and disadvantages of non-invasive malaria diagnostics. It discusses perceived benefits such as ease of use, reduced pain, and increased screening capabilities as well as potential challenges and limitations such as the inability to differentiate parasite species and logistical concerns.

Reviewer #2: GENERAL REMARKS
In this manuscript, the authors present a relevant qualitative study that aimed to generate evidence from end-users around the potential adoption of non-invasive diagnostic technologies, to determine whether these tools are fit for purpose. To this end, they conducted, in three malaria-endemic countries (Indonesia, Peru, and Rwanda), 24 semi-structured interviews with stakeholders and professionals working at borders, and 16 focus group discussions with community members, caregivers of children under 5 years, pregnant women, healthcare workers, and teachers (total n=139). The participants shared their practical needs, concerns, and acceptability regarding the proposed non-invasive tools. Therefore, the study provides valuable insights and guidance for the development of non-invasive malaria diagnostics, ensuring that these tools meet the needs of end-users. Despite the relevance of this study, the authors should address some minor points before the publication of the manuscript.

- We would like to thank both reviewers for their encouraging and positive feedback on our study and manuscript. We have tried to address the majority of the minor comments raised by Reviewer 2.

  MINOR ISSUES

  Introduction
  • Several sentences lack references (e.g., lines 56, 61, 90, 95, 98, 99, 106). Please carefully review the introduction and add appropriate citations where needed. Thanks for your suggestion, 5 more references have been added.
  • Lines 76–77: Clarify what UNITAID and FIND are. Addressed

  Materials and Methods
  • Line 147: Please explicitly define “stakeholders” (e.g., specify roles like policymakers, NGO representatives, or ministry officials). While contextualized later, a clear definition early would aid readability. Thank you for this suggestion. We had this address, but it was not clear enough. We have included in brackets the specific stakeholders within that sentence (Line 158 to 160).
  • Line 150: I recommend clarifying the definition of “community members”. Would they be anyone in the community who is not from the other groups (pregnant women, teachers, caregivers of children under five, and healthcare workers)? Indeed, this needed to be defined better. We added “other” to the term “community members” (Line 163 to 168). This classification included other members of the community who do not fall into the above categories but who are interested in sharing their opinions on the subject. Selection of community members was decided with partners. They could include parents and school staff (recruited at schools), travellers (recruited at travel agencies), established groups at the community (including spiritual/healthcare centres etc.). We have included some wording throughout the manuscript.
  • Lines 147–151: Explain the criteria used to establish which method (Semi-Structured Interviews or Focus Group Discussions) was more appropriate for each listed group (e.g., stakeholders, professionals working at borders, community members, pregnant women, teachers). Semi-structured interviews were chosen when the objective was to capture in-depth, individual perspectives on context-specific issues, and when engaging with professionals who might not have the time to participate in a group discussion but could accommodate some time for an individual conversation (e.g., stakeholders, professionals working at borders). In contrast, focus group discussions were used when the aim was to explore shared experiences, collective norms, and group dynamics (e.g., community members, teachers, pregnant women), as this method encouraged interaction and exchange among participants from the same community groups. Thanks to the reviewer this has been clarified within the manuscript (lines 154 to 157).
  • Lines 156–163: Justify differences in the endemicity levels and standardize their characterization across the selected areas in the three countries. For instance: Indonesia had no sampling from non-endemic areas, unlike Rwanda and Peru. Peru only sampled highly endemic areas, while Indonesia included low-, medium-, and high-endemicity areas. For Rwanda the endemic areas are mentioned without specifying the endemicity level. Thanks for this suggestion. We have now included a table to categorize better study locations across countries (Table 1). Not all locations (rural, urban, malaria endemic, and non-endemic) in the three countries had participants available for SSI/FGD in all types of areas. In addition, limited resources restricted the sampling to the ones that were more relevant for country context. We have clarified this in the revised manuscript (Lines 156–163).

• Lines 156–163: The selected areas in Indonesia and Rwanda lack description as urban or rural, while those in Peru are clearly characterized. I recommend standardizing this classification across all study sites for better comparability. Thanks for this suggestion. As stated above, we have now included in a table to categorize better study locations across countries (Table 1). (Lines 156–163).

• Lines 156–163: I suggest to present site characteristics (including FGD/SSI counts and sample sizes) in a table for a better overview of the sampling. Thanks to this suggestion, we have included a Table within the Methods section to explain the sampling procedures.

• Line 162: Fix spacing in "and Tumbes". Addressed

• Line 164: What is the reason for discussing Peru-specific findings separately and excluding them from this manuscript? Our partners in Peru were highly motivated to publish a manuscript focused specifically on the findings from the Peruvian context (Nussbaum, L., Ortega, E., Ríos López, E.J. *et al.* Voices from the Amazon: exploring implementor and user perceptions of non-invasive malaria diagnostics in Peru. *Malar J* **24**, 32 (2025). https://doi.org/10.1186/s12936-025-05273-1). While those data are also included in the current submission, the focus of this manuscript is broader, presenting a comparative analysis across three countries. In contrast, the Peru-focused publication offers an in-depth examination of local findings unique to that setting. Partners in Indonesia are already preparing another separate country-focused manuscript to explore in depth country context perceptions.

• Line 180: Fix spacing in "aged 18 years". Addressed

• Line 252: I recommend sharing SSI and FGD guides as supporting information for more transparency of the applied methods. Thanks for this suggestion. We have included the guides as supporting information.

• Lines 259–261: Was the information in Figure S1 the only explanation given to participants about the devices? Was the figure translated into local languages? Were the devices limitations or additional details provided? The devices were presented following the SSI and FGD guides. We have now included that information as Supporting information. All the information presented to participants was shown in their local languages.

• I suggest reporting how many people refused to participate or dropped out of the study. We appreciate the reviewer’s suggestion. Unfortunately, as this was a qualitative exploratory study, we did not systematically track the number of potential participants who were eligible or who refused to take part in the study. There was no follow up, thus, no drop out participants. Participants provided consent and participated on the SSIs or FGDs.

Results

• Explain why according to Table 1 Indonesia had no community members included. In Indonesia we didn’t include ‘other community members’ mainly because of practical reasons. The study had to be completed within a limited timeline, and with the geographical spread of our sites it was already challenging, there were travel constraints and, in some areas, no daily flights, which made scheduling difficult. Given this, we prioritized groups most directly relevant to the study objectives since they were the ones most closely connected to the use and implementation of the non-invasive malaria testing. While perspectives from the broader community would have been valuable, they were considered less critical for this phase of the study.

• I recommend the addition of relative values (percentages) and a "Total" column in Table 1 for better interpretation. Thank you for your comment, we have updated the Table.

• Lines 296–267: The authors mention “the interviewer remaining neutral and unbiased throughout”. Please clarify their stance on answering the questions of participants about the devices. If questions were answered, this should be explicitly stated. We thank the reviewer for highlighting this point. The interviewers were trained to maintain neutrality and avoid leading the participants to specific responses. However, they did provide clarifications about the devices when participants asked for explanations.

• Figure 2: Correct the misspelling of "malaria" in "Perceptions that malaria can only be detected in blood". Addressed, a revised version of Fig2 has been uploaded.

Discussion
• While the study focuses on participants’ perceptions, discussing whether these perceptions align with scientific evidence (especially the perceived disadvantages) would strengthen the paper. The authors are not required to do this modification, but it could add valuable context. We sincerely appreciate the reviewer’s suggestion. At present, there is unfortunately very limited scientific literature on this specific topic. We have included the two references we were able to identify and briefly discussed one of them in the manuscript (lines 861–864). Within the limits of the available evidence, we feel this is the most we can incorporate at this stage, but we agree that further studies in this area would add valuable context.

ADDITIONAL POINT
• Regarding data availability: While the authors state that “all data are fully available without restriction”, I was unable to locate the interview transcripts or excerpts of the transcripts relevant to the study in the Supporting Information files. We thank the reviewer; this was a mistake during the submission process. We have changed this to the statement: "The protocol for this study, approved by the Ethics Committee, did not include provisions for the public sharing of the full database generated during the study. As participants did not provide consent to share their data publicly and to ensure compliance with ethical guidelines, the authors did not include the full database in a public repository. The datasets used and/or analyzed during the current study are available from the corresponding author on reasonable request or at the following email address: [info@finddx.org](mailto:info@finddx.org).” To maintain participants' anonymity, we keep the transcripts secured. By direct contact with the first author, we will be able to share the full data upon reasonable request.

6. PLOS authors have the option to publish the peer review history of their article ([what does this mean?](https://track.editorialmanager.com/CL0/https:%2F%2Fjournals.plos.org%2Fplosone%2Fs%2Feditorial-and-peer-review-process%23loc-peer-review-history/1/010f0197a1ee0a23-7c5f961b-1d73-43b3-bcd4-b30bc4ac3907-000000/2vGXOJG3hY2RyQaA-XZYtPt2Tlr2Ace2449i8VoLd4w=216)). If published, this will include your full peer review and any attached files.

If you choose “no”, your identity will remain anonymous but your review may still be made public.

**Do you want your identity to be public for this peer review?** For information about this choice, including consent withdrawal, please see our [Privacy Policy](https://track.editorialmanager.com/CL0/https:%2F%2Fwww.plos.org%2Fprivacy-policy/1/010f0197a1ee0a23-7c5f961b-1d73-43b3-bcd4-b30bc4ac3907-000000/pjt5gh_ZGv2Qd451HCponedHrDlK2CkWxocrBSeEAAY=216).

Reviewer #1: No

Reviewer #2: **Yes:** Laura Cordeiro Gomes
