# Supplementary material for: “It doesn’t require blood!”: Perceptions around non-invasive malaria testing tools in Indonesia, Peru, and Rwanda
Source: PLoS One. 2026 Jan 9;21(1):e0318393. doi: 10.1371/journal.pone.0318393 (PMC12788683; doi:10.1371/journal.pone.0318393)
Supplement: Attachment — Submitted filename: Response to reviewer.docx [file pone.0318393.s007.docx]

Additional Editor Comments :

The line numbers below correspond to the “track changes” version.
Line 80: I don’t believe it is essential to describe UNITAID in detail, can omit the explanatory sentence. Thanks, we addressed this by removing the sentence.

Line 82: where is the reference to the technical landscape assessment by FIND? We are currently updating the document and plan to submit it to a peer-reviewed journal. At this stage, however, the document remains internal and unpublished. For this reason, when referring to the UNITAID landscape we use the term *“published”*, while for the FIND landscape, we use *“conducted by FIND”*. We hope this clarification is clear.

Line 159: suggest small rewrite “and professionals working at borders who, because of human mobility, were familiar with malaria policies”. Thanks for the suggestion, we reviewed accordingly.

Line 210: Why do border professionals appear both in row 2 and row 3 of Table 2. Should they not appear in only one row? We apologize to the reviewer, but we don’t see “border professionals” appearing twice; maybe is a formatting issue from the track change version.

Line 238: Suggest “These are…” not “Are”. This point has been addressed at row 226 of the revised tracked-changes document.

Line 307: suggest amend sentence to say “there were 7 FGDs in Peru, 5 in Rwanda and 4 in Indonesia”. The total numbers of participants appear in the table and do not need to be repeated in the text. This point has been addressed at row 295 of the revised tracked-changes document.

Line 416: How were the interviewees made aware of the characteristics of the new tests, in particular the fact that they could not differentiate different species of malaria parasites? Were they shown Figures 1 and 2, or how was this information transmitted? Figure S1, included in the supplementary information, was used to guide the discussion and provide an illustration of how the devices might look. Additional details, such as their capacity to differentiate between malaria species, were conveyed orally during the interviews within participants questions, answers and discussion. In S3 Guide for the FGDs you can see how the question regarding the parasite species was asked “What are the key characteristics that each instrument shall have? (List key characteristics quoted by each participant and check if the ability to differentiate parasite species emerged. If not, ask the question: Do you think it would be relevant if the instrument can differentiate between parasite species (Vivax, Falciparum, Ovale, Malaria, knowlesi)?” for participants' exploration.

Line 642-651: I find this paragraph unnecessarily detailed, especially since there is no similar paragraph for Rwanda and Peru. Perhaps rephrase: “In Indonesia, participants highlighted the importance of Puskesmas (primary health centers), community health centers, and community health cadres (volunteers)”. This structure is very common in many low- and middle-income countries. Thanks for this suggestion, we have reviewed the text to be less specific. Now it reads: “In Indonesia, participants highlighted the importance of primary health centers, that offer a wide range of basic healthcare services, community health centers that serve as the first point of contact for most, and health cadres with authorized volunteers to administer malaria medication, especially in remote areas.”
